# The Impact of Oxidative Stress on Adipose Tissue Energy Balance

**DOI:** 10.3389/fphys.2019.01638

**Published:** 2020-01-22

**Authors:** Peter M. Masschelin, Aaron R. Cox, Natasha Chernis, Sean M. Hartig

**Affiliations:** ^1^Division of Endocrinology, Diabetes, and Metabolism, Department of Medicine, Baylor College of Medicine, Houston, TX, United States; ^2^Department of Molecular and Cellular Biology, Baylor College of Medicine, Houston, TX, United States

**Keywords:** adipocyte, metabolism and obesity, oxidative stress, mitochondria, mitochondrial disorders

## Abstract

Overnutrition and sedentary activity reinforce the growing trend of worldwide obesity, insulin resistance, and type 2 diabetes. However, we have limited insight into how food intake generates sophisticated metabolic perturbations associated with obesity. Accumulation of mitochondrial oxidative stress contributes to the metabolic changes in obesity, but the mechanisms and significance are unclear. In white adipose tissue (WAT), mitochondrial oxidative stress, and the generation of reactive oxygen species (ROS) impact the endocrine and metabolic function of fat cells. The central role of mitochondria in nutrient handling suggests pharmacological targeting of pathological oxidative stress likely improves the metabolic profile of obesity. This review will summarize the critical pathogenic mechanisms of obesity-driven oxidative stress in WAT.

White adipose tissue (WAT) is an endocrine organ that stores energy in the form of lipids and secretes hormones essential for insulin sensitivity and energy homeostasis. The fat cell interprets nutritional, hormonal, and sympathetic tone in the tissue microenvironment to store and liberate fuels until whole-body energy demands necessitate fatty acid liberation. Like other cells, mitochondria in adipocytes assimilate signals that reflect the energy status of the cell and produce the majority of ATP from macronutrients through cellular respiration and oxidative phosphorylation (OXPHOS).

Obesity engenders nutrient stress that stifles mitochondrial capacity to sustain ATP levels in response to energy demands (Bournat and Brown, 2010; Liesa and Shirihai, 2013). Elevated mitochondrial substrate load consequently increases electron transport chain (ETC) activity and reactive oxygen species (ROS) production. Obese individuals exhibit higher levels of oxidative stress in WAT, including elevated ROS levels and decreased antioxidant activity coupled with alterations in adipokines required for insulin sensitivity (Furukawa et al., 2004). Moreover, oxidative stress associates with intra-abdominal obesity and insulin resistance (Furukawa et al., 2004; Frohnert et al., 2011). These results indicate the oxidizing environment in WAT of obese individuals likely impacts fat cell function and energy balance. Numerous questions remain, including how gradients of ROS inside the cell impact signaling cascades and gene regulation.

## Nutrient Imbalance Provokes Mitochondrial Ros

**FIGURE 1:**
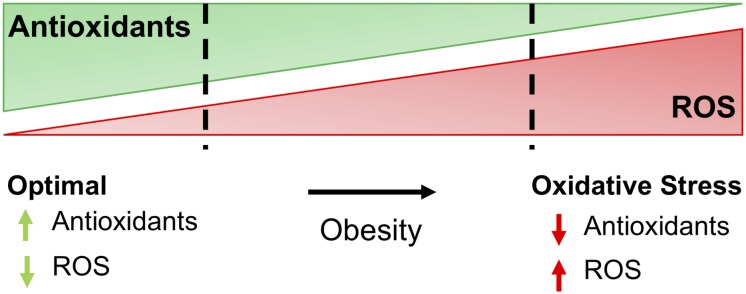
The balance of antioxidants and ROS determine oxidative stress. For most cells, optimal redox conditions are achieved when higher levels of antioxidants are present to quench reactive oxygen species (ROS), maintaining ROS at low levels. Obesity and comorbidities increase ROS and decrease antioxidants in adipose, leading to oxidative stress and further complications of obesity, including insulin resistance and diabetes.

Oxidative stress represents a disturbance in the equilibrium of ROS production and antioxidant defenses (Figure 1). At the molecular level, ROS mainly emerge from the mitochondrial ETC (Starkov, 2008; Murphy, 2009). Electron transfer through the ETC generates superoxide anions as byproducts, with complex I and III representing primary sources of ROS. Under certain conditions, complex II and other cellular ROS sources can contribute to the overall pool. Superoxide is the primary ROS species that reacts with Fe-containing proteins to generate H_2_O_2_. H_2_O_2_ accumulation in the cell contributes directly to the metabolic imbalance linking excessive nutrient stress and insulin resistance (Anderson et al., 2009; Akl et al., 2017; Fazakerley et al., 2018). However, ROS also encompass a diverse range of chemical entities, including nitric oxide, peroxynitrite, hypochlorous acid, singlet oxygen, and the hydroxyl radical. Consequently, the broad biological impacts of ROS derive from multiple cell and tissue microenvironments that divide physiological and pathological effects.

The conditions that favor mitochondrial superoxide production include reduction of electron carrier pools associated with the mitochondrial respiratory chain (NADH, flavins, ubiquinone), a high proton motive force, and elevated oxygen consumption within the mitochondria (Murphy, 2009). Overnutrition supplies excess electrons to the respiratory chain, while lack of physical activity and low ATP demand favors a high proton motive force with a low respiration rate, leading to mitochondrial superoxide formation and oxidative stress. By contrast, in mitochondria actively making ATP, superoxide production is low because the electron carriers are relatively oxidized, the proton motive force is small, and the respiration rate is high.

Prolonged oxidative stress directly impacts metabolism, including the activity of enzymes involved in the TCA cycle and the ETC (Quijano et al., 2016). The TCA cycle enzyme aconitase catalyzes the interconversion of citrate and isocitrate to regulate the availability of intermediates for lipid synthesis and ATP production. Citrate is the last common metabolite on the pathways for oxidation of acetyl-CoA and its export for fatty acid synthesis in the cytoplasm. Superoxide inhibits aconitase (Hausladen and Fridovich, 1994; Gardner et al., 1995), leading to diversion of acetyl-CoA away from OXPHOS and toward fat storage (Armstrong et al., 2004; Lushchak et al., 2014). This feedback loop may be part of an antioxidant defense mechanism that adapts prolonged mitochondrial superoxide production (Scandroglio et al., 2014). Acetyl-CoA diversion may slow delivery of electron carriers such as NADH to the respiratory chain, thereby decreasing ROS production (Armstrong et al., 2004).

Oxidative stress also impacts pyruvate dehydrogenase kinase 2 (PDK2) inhibition of the pyruvate dehydrogenase complex (PDC) (Hurd et al., 2012) and fat metabolism. ROS oxidize critical cysteine residues, disabling PDK2, and supporting acetyl-CoA synthesis from glucose-derived pyruvate. Therefore, elevated mitochondrial superoxide and H_2_O_2_ couples PDC activity with aconitase interruption to divert citrate from the TCA cycle to the cytoplasm as triglycerides during overnutrition. These studies suggest persistent nutrient stress impairs the physiological behavior of crucial metabolic enzymes needed for balanced ATP generation and consumption.

## Antioxidant Response to Mitochondrial Ros Regulates Adipose Tissue Function

A variety of peroxidases, including catalase, glutathione peroxidases, and peroxiredoxins (Prdxs) that control the levels of H_2_O_2_ in the cell and protect against ROS-induced damage by catalyzing the reduction of H_2_O_2_ into water. Along these lines, overexpression of catalase (Anderson et al., 2009; Barbosa et al., 2013; Lee et al., 2017; Paglialunga et al., 2017) or other methods that block H_2_O_2_ generation (Anderson et al., 2009; Boden et al., 2012) preserve insulin sensitivity in cell models and rodents fed high-fat diet.

The mitochondrial antioxidant peroxiredoxin 3 (Prdx3) responds to oxidative stress and scavenges H_2_O_2_. Levels of Prdx3 are decreased in obese humans and mice, potentially contributing to oxidative stress intolerance (Huh et al., 2012). Whole-body deletion of *Prdx3* in mice causes obesity and increased expression of lipogenic genes in adipocytes, while decreasing expression of lipolytic genes. As a result, hypertrophic adipocytes exclusively accumulate excess lipids and cannot enable appropriate energy balance control. In addition to altering the balance of lipogenesis and lipolysis, Prdx3-deficient adipocytes exhibited increased superoxide production, decreased mitochondrial potential, and altered adipokine expression, including decreased adiponectin.

Okuno et al. (2018) created “Fat ROS-augmented” (AKO) and “Fat ROS-eliminated” (aP2-dTg) mice to address the question of how ROS affect WAT function. AKO mice leverage adipocyte-specific ablation of glutamate-cysteine ligase (*Gclc*) to disable the rate-limiting step in glutathione synthesis and increase ROS generation. AKO mice fed high fat/high sucrose (HF/HS) diet for 6 weeks had smaller adipocytes and decreased expression of lipogenic genes, including *Acly*, *Scd1*, *Fasn*, *Acaca*, and *Srebf1*. Insulin sensitivity was also reduced. Conversely, mice expressing rat catalase and human SOD1 under the aP2 promoter had the opposite phenotype. These mice (aP2-dTg) showed reduced H_2_O_2_ in subcutaneous and gonadal WAT. Feeding a HF/HS diet yielded beneficial subcutaneous and gonadal WAT expansion mirrored by increased expression of lipogenic genes (*Acly*, *Scd1*, *Fasn*, and *Acaca*) and insulin sensitivity.

While these data argue that increasing mitochondrial antioxidants protects against oxidative stress in WAT, genetic alteration of other mitochondrial antioxidants reveal different phenotypes. Manganese superoxide dismutase (MnSOD) is an important mitochondrial antioxidant that detoxifies superoxides (Holley et al., 2011). Adipocyte-specific knockout of MnSOD protected against diet-induced WAT expansion and weight gain (Han et al., 2016). Mechanistically, MnSOD knockout in adipocytes triggered an adaptive stress response that activated mitochondrial biogenesis and enhanced mitochondrial fatty acid oxidation, thereby preventing diet-induced obesity and insulin resistance. Increased ROS levels correlated with Uncoupling Protein 1 (UCP1) activation in subcutaneous WAT and higher energy expenditure (Han et al., 2016). These disparate features of mice that lack the *Prdx3* and *MnSOD* genes coupled with therapeutic shortcomings of antioxidant therapies in human clinical trials (Fusco et al., 2007; Bjelakovic et al., 2013, 2014) suggest a more complex interaction of metabolism and redox balance in WAT.

## Mitochondrial Redox Reactions Generate Divergent Inputs for Cellular Signaling

The homeostatic systems that regulate oxidative stress in the lean state are largely repressed in obesity due to the accumulation of oxidized biomolecules within WAT. Excessive ROS irreversibly damages DNA, lipids, and proteins with adverse effects on cellular functions. Increased oxidative stress can alter proteins and lipids through direct and indirect pathways that culminate in oxidation of side chains and lipid-protein adduction (Grimsrud et al., 2008; Davies, 2016).

Reactive oxygen species oxidation of lipids ultimately generates lipid aldehydes that modify DNA, proteins, RNA, and other lipid species (Esterbauer et al., 1991; Uchida, 2003). Increased markers of lipid peroxidation, including thiobarbituric acid reactive substances (TBARS) and 8-epi-prostaglandin-F2α (8-epi-PGF2α) are observed in individuals with higher BMI and waist circumference (Furukawa et al., 2004). Oxidized lipids and proteins preferentially accumulate in visceral depots compared to subcutaneous depots of obese mice (Long et al., 2013; Hauck et al., 2018, 2019) and humans (Frohnert et al., 2011), suggesting ROS modifications correlate with conditions associated with type 2 diabetes, including central fat accrual.

Highly reactive hydroxyl radicals (⋅OH) can be generated when excess H_2_O_2_ reacts with ferrous iron. Unlike H_2_O_2_, ⋅OH cannot undergo detoxification. Instead, ⋅OH removes electrons from neighboring lipids, proteins, and nucleic acids. Lipid aldehydes are highly electrophilic and prone to irreversible nucleophilic attack by the side chains of lysine (Lys), histidine (His), and cysteine (Cys) residues of proteins, resulting in a covalent lipid-protein adduct termed protein carbonylation (Schaur, 2003; Curtis et al., 2012). Furthermore, Lys, His, and Cys residues often cluster within active sites of enzymes or critical structural motifs, so their stable modification by lipids generally leads to inhibition or deactivation of protein function. However, recent work challenges the notion that ROS-driven modifications broadly degrade fat cell function. Brown adipose tissue (BAT) contains elevated levels of mitochondrial superoxide, mitochondrial H_2_O_2_, and oxidized lipids that correlate with acute activation of thermogenesis (Chouchani et al., 2016a, b). Mitochondrial ROS in BAT can converge on UCP1 C253 inducing cysteine sulfenylation (-SOH) (Chouchani et al., 2016a). Interestingly, UCP1 C253A does not disable thermogenic responses in brown adipocytes but desensitizes the protein to adrenergic activation of uncoupled respiration. Further exploration of physiological ROS signaling outputs and modifications may show how redox status in adipocytes contributes to energy balance.

Polyunsaturated fatty acids (PUFAs) are abundant in WAT and particularly sensitive to lipid peroxidation. One major consequence of lipid peroxidation is mitochondrial membrane damage (Kowaltowski and Vercesi, 1999). Also, peroxidation of PUFAs results in the release of diffusible reactive lipid aldehydes. Among the wide variety of reactive lipids formed through this mechanism, 4-hydroxy-non-enal (4-HNE) derived from oxidation of n6 fatty acids and 4-hydroxy-hexenal (4-HHE) from n3 fatty acid oxidation are the most widely studied in the context of adipose biology. The WAT of obese mice showed decreased metabolism of 4-HNE, while stress response proteins, including glutathione-S-transferase M1, glutathione peroxidase 1, and Prdx (Grimsrud et al., 2007) were carbonylated. Lipid peroxidation end products can also inhibit insulin signaling as 4-HNE de-stabilizes IRS adapter proteins and insulin receptor β (Demozay et al., 2008; Frohnert and Bernlohr, 2013).

Lipid peroxidation products also damage the function of transcription factors that contain zinc-finger motifs, histones, and other nuclear proteins of visceral fat cells isolated from obese mice (Hauck et al., 2018). The lipid peroxidation of transcriptional regulatory proteins presents a consolidated mechanism for retrograde ROS signaling from mitochondria to the nucleus. Although mitochondria are the most significant source of ROS, the discovery of lipid-protein adducts in the nucleus of adipocytes suggests either a different pool of ROS contributes to lipid peroxidation or a mechanism exists to sequester and shuttle reactive aldehydes to specific subcellular localizations (Hauck et al., 2018). As with ROS, the timing of protein carbonylation may be important for beneficial or pathologic effects. Acute carbonylation of substrates after exercise are potentially beneficial, while chronic accumulation of carbonylated proteins in the muscle and WAT of obese and sedentary individuals may be pathological and contribute to comorbidities of obesity (Frohnert and Bernlohr, 2013).

Additionally, ROS seem to be important in the cellular aspects of adipocyte differentiation. Numerous studies demonstrate that mitochondrial biogenesis increases during adipocyte differentiation (Wilson-Fritch et al., 2003; Lu et al., 2010; Zhang et al., 2013). Dramatic expansion of mitochondrial content enables higher metabolic rates to overcome the energetic demands of differentiation. Induction of differentiation correlates with superoxide generation from complex III, conversion of superoxide to H_2_O_2_, and activation of transcriptional machinery necessary for adipogenesis (Tormos et al., 2011). Mechanistically, ROS production in differentiating cells coincides with increased C/EBPβ binding to DNA and accelerated mitotic clonal expansion (Kim J.W. et al., 2007; Lee et al., 2009). However, obesity-mediated ROS induction also restricts mitochondrial biogenesis and adipocyte differentiation. Higher accumulation of 4-HNE adducts occurs in cultured differentiating preadipocytes from insulin-resistant compared to insulin-sensitive individuals. In this manner, treatment of primary subcutaneous preadipocytes from obese individuals with pathological levels of 4-HNE decreased markers associated with insulin sensitivity and mature fat cells (Dasuri et al., 2013; Elrayess et al., 2017). Other studies demonstrate that treatment with antioxidants decreases differentiation (Tormos et al., 2011) and disrupts UCP1-dependent thermogenic responses (Ro et al., 2014; Chouchani et al., 2016a). Divergent *in vitro* and *in vivo* findings illustrate existing challenges in defining the specifics of ROS signaling and its connectivity to metabolic diseases.

## Oxidative Stress Contributes to the Comorbidities of Metabolic Diseases

Nutrient overload has been linked to the development of insulin resistance. In a pioneering study, healthy men fed ∼6000 kcal/day for 1 week exhibited WAT insulin resistance and oxidative stress in addition to protein oxidation and carbonylation (Boden et al., 2015). One carbonylated protein of importance was GLUT4, whose carbonylation likely impairs insulin-stimulated glucose uptake. Of note, systemic oxidative stress and insulin resistance did not coincide with inflammatory cytokines in plasma nor ER stress in WAT. These findings provide a causal link between oxidative stress and insulin resistance in humans.

Mitochondrial metabolism is often altered in inherited diseases, such as inborn errors of metabolism (IEMs) that impinge upon ROS generation. Inhibition of OXPHOS increases ROS generation due to a backlog of electrons in the various complexes, resulting in electron leak, ROS generation, and production of H_2_O_2_. In IEMs affecting the ETC or other pathways of ATP generation, increased oxidative stress is often observed, while the exact mechanisms for increased ROS production are unknown. It is hypothesized that mutations affecting the formation of the protein complexes in the ETC or mutations that modify their assembly increase ROS generation by facilitating electron leak (Olsen et al., 2015). Additionally, accumulation of toxic intermediates, often observed in IEMs, can increase the ROS generation by further decreasing OXPHOS activity, as in the case of medium-chain acyl-CoA dehydrogenase (MCAD) deficiency. MCAD deficiency reflects the accumulation of medium-chain fatty acid derivatives, including cis-4-decenoic acid, octanoate, and decanoate, with these metabolites altering levels of antioxidants and increasing markers of oxidative stress (Schuck et al., 2007, 2009). Intriguingly, IEMs display metabolic reprograming with a switch to glycolysis for both ATP production and muted ROS generation (Olsen et al., 2015). Specifically, in myoclonic epilepsy with ragged red fibers (MERRF), increased intracellular H_2_O_2_ levels correspond with increased AMPK phosphorylation and expression of GLUT1, hexokinase II, and lactate dehydrogenase. These results, as well as increased lactic acid production, all point to increased glycolysis (De la Mata et al., 2012; Wu and Wei, 2012). In multiple acyl-CoA dehydrogenase deficiency (MADD), mutations in *ETFa*, *ETFb*, or *ETFDH*, lead to decreased ATP production with an accumulation of organic acids, including glutaric acid as well as acyl-carnitines. A subset of these patients is riboflavin responsive (RR-MADD) with high dose riboflavin alleviating some symptoms. Similar to MERRF, many RR-MADD patients exhibit increased oxidative stress (Cornelius et al., 2013, 2014). This defect may be due to defective electron transfer and increased electron leak from the misfolded ETFDH protein and decreased binding of CoQ10 (Cornelius et al., 2013). Treatment with CoQ10, but not riboflavin, decreased ROS levels (Cornelius et al., 2013). Analysis of mitochondrial function from RR-MADD fibroblasts showed increased mitochondrial fragmentation and reduced β-oxidation, while supplementation with the antioxidant CoQ10 decreased fragmentation and mitophagy (Cornelius et al., 2014). While obesity and IEMs are distinct disorders, both conditions impinge on energy balance in WAT. Even though these disorders have very different manifestations, oxidative stress plays an important role in both and may be a therapeutic target. For example, CoQ10 is often given as a broad-spectrum treatment to individuals with IEMs, and while its effectiveness is debated, the anti-inflammatory effects may be beneficial in reducing oxidative stress and the pathogenesis of the disease (Cornelius et al., 2013; Acosta et al., 2016; Zhai et al., 2017).

## Leveraging Redox Balance to Improve Insulin Sensitivity

Mitochondria represent control centers of many metabolic pathways. Interventions that enhance adipocyte mitochondrial function may also improve whole-body insulin sensitivity. Mitigation of mitochondrial ROS production and oxidative stress may be a possible therapeutic target in type 2 diabetes and IEMs because some mitochondrial-targeted antioxidants and other small molecule drugs improve metabolic profiles in mouse models (Feillet-Coudray et al., 2014; Fouret et al., 2015; Rivera-Barahona et al., 2017) and human studies (Escribano-Lopez et al., 2018).

Thiazolidinediones (TZDs) are PPARγ agonists used for treating type 2 diabetes (Kelly et al., 1999; King, 2000; Khan et al., 2002; Goldberg et al., 2005; Deeg et al., 2007). TZDs, such as rosiglitazone and pioglitazone, enhance insulin sensitivity by improving adipokine profiles (Maeda et al., 2001, 2002) and reducing fasting blood glucose levels (Boyle et al., 2002; Chappuis et al., 2007). TZDs also promote insulin sensitivity by directing fatty acids to subcutaneous fat, rather than visceral fat. Subcutaneous fat expandability, even in the context of obesity and type 2 diabetes, correlates with insulin sensitivity in rodents and humans (Ross et al., 1996; Miyazaki et al., 2002; Kim J.Y. et al., 2007; Tran et al., 2008; Porter et al., 2009). Numerous *in vitro* and *in vivo* studies demonstrate TZDs enhance mitochondrial biogenesis, content, function, and morphology. Rosiglitazone also induces cellular antioxidant enzymes responsible for the removal of ROS generated by increased mitochondrial activity in adipose tissue of diabetic rodents (Rong et al., 2007) and humans (Bogacka et al., 2005; Rong et al., 2007; Ahmed et al., 2010). It is now well established that anti-diabetic PPARγ agonists also activate a BAT gene program in white adipocytes, converting them to “beige” cells that express UCP1 (Tiraby et al., 2003; Wilson-Fritch et al., 2003; Bogacka et al., 2005; Ohno et al., 2005; Petrovic et al., 2010). Taken together, TZDs impact WAT mitochondrial function in multiple ways that ultimately improve systemic fat metabolism and insulin sensitivity. Other therapeutic strategies include mitochondria-targeted scavengers (Smith et al., 2012) and chemical uncouplers that dissipate energy as heat (Perry et al., 2013; Goldgof et al., 2014). However, these methods to enhance mitochondrial function display a narrow therapeutic range that limits safe use for obesity.

Although the development of insulin resistance does not require impaired mitochondrial function (Hancock et al., 2008; Holloszy, 2013), pathways promoting insulin resistance may impair mitochondrial function and further increase ROS production, resulting in a detrimental feedback loop. Aerobic exercise and caloric restriction disrupt this vicious loop, potentially by preventing accumulation of injured mitochondrial proteins with substantial improvement of insulin sensitivity. In insulin-resistant people, aerobic exercise stimulates both mitochondrial biogenesis and efficiency concurrent with an enhancement of insulin action (Mul et al., 2015). Ultimately, exercise engages pathways that reduce ROS coupled with insulin sensitivity and improved mitochondrial function in WAT.

## Conclusion

**FIGURE 2:**
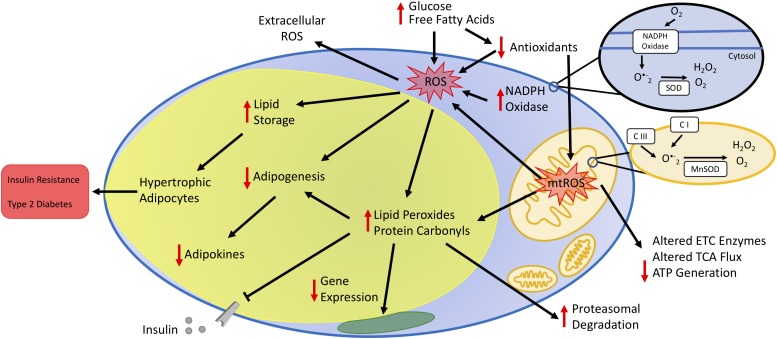
Impact of oxidative stress on adipocyte function. Increased plasma glucose and free fatty acids contribute to increased oxidative stress by increasing the production of reactive oxygen species (ROS) and decreasing antioxidant concentrations. Increased oxidative stress occurs via enzymes in the cytoplasm, such as NADPH oxidase, and the mitochondria. The oxidative environment increases lipid storage resulting in hypertrophic adipocytes. Additionally, increased mitochondrial ROS (mtROS) alters the activity state of metabolic enzymes either directly or by changing the oxidative state of protein side-chains or by other post-translational modifications, including lipid peroxidation and protein carbonylation. Cumulatively, increased adipocyte oxidative stress decreases adipogenesis and secretion of adipokines, leading to unbalanced energy homeostasis, insulin resistance, and type 2 diabetes.

Obesity is the result of excessive expansion of WAT depots due to a chronic imbalance between energy intake and expenditure. Many studies demonstrate that oxidative stress in fat cells links obesity and its comorbidities. The fact that WAT remains the sole organ for storing surfeit lipid renders the macromolecules in adipocytes particularly vulnerable to carbonylation and other modifications driven by oxidative stress. Prolonged oxidative stress negatively influences endocrine and homeostatic performance of WAT, including disruption of hormone secretion, elevation of serum lipids, inadequate cellular antioxidant defenses, and impaired mitochondrial function (Figure 2). Metabolic challenges, such as persistent nutrient intake and sedentary behaviors that promote impaired glucose and lipid handling, also elevate mitochondrial ROS production to cause adipocyte dysfunction. Consequently, adipocytes cannot engage appropriate transcriptional and energetic responses to enable insulin sensitivity.

The increasing prevalence of obesity suggests lifestyle intervention as the principal method to treat obesity is unlikely to succeed. Currently, all available anti-obesity medications act by limiting energy intake through appetite suppression or inhibition of intestinal lipid absorption. However, these medications are largely ineffective and often have adverse side effects. The central role of mitochondria in nutrient handling provides a logical entry point for improving metabolism in obesity. While approaches to understanding and intervening in oxidative damage evolve, exploration of mitochondria redox balance may enable development of dietary and small molecule therapies for obesity and its comorbidities.

## Author Contributions

PM, AC, NC, and SH wrote the manuscript. SH secured funding.

## Conflict of Interest

The authors declare that the research was conducted in the absence of any commercial or financial relationships that could be construed as a potential conflict of interest.
